# Silicon Nitride-Based Micro-Apertures Coated with Parylene for the Investigation of Pore Proteins Fused in Free-Standing Lipid Bilayers

**DOI:** 10.3390/membranes12030309

**Published:** 2022-03-09

**Authors:** Tanzir Ahmed, Jayesh Arun Bafna, Roland Hemmler, Karsten Gall, Richard Wagner, Mathias Winterhalter, Michael J. Vellekoop, Sander van den Driesche

**Affiliations:** 1 Institute for Microsensors, -actuators and -systems (IMSAS), University of Bremen, D-28359 Bremen, Germany; tahmed@imsas.uni-bremen.de (T.A.); mvellekoop@imsas.uni-bremen.de (M.J.V.); 2 Department of Life Sciences and Chemistry, Jacobs University, D-28759 Bremen, Germany; j.bafna@jacobs-university.de (J.A.B.); ri.wagner@jacobs-university.de (R.W.); m.winterhalter@jacobs-university.de (M.W.); 3 Ionovation GmbH, D-49143 Bissendorf, Germany; roland.hemmler@ionovation.com (R.H.); karsten.gall@ionovation.com (K.G.)

**Keywords:** silicon nitride aperture, Parylene-C, Parylene-AF4, bilayer lipid membrane (BLM), outer membrane vesicle (OMV)

## Abstract

In this work, we present a microsystem setup for performing sensitive biological membrane translocation measurements. Thin free-standing synthetic bilayer lipid membranes (BLM) were constructed in microfabricated silicon nitride apertures (<100 µm in diameter), conformal coated with Parylene (Parylene-C or Parylene-AF4). Within these BLMs, electrophysiological measurements were conducted to monitor the behavior of different pore proteins. Two approaches to integrate pore-forming proteins into the membrane were applied: direct reconstitution and reconstitution via outer membrane vesicles (OMVs) released from Gram-negative bacteria. The advantage of utilizing OMVs is that the pore proteins remain in their native lipid and lipopolysaccharide (LPS) environment, representing a more natural state compared to the usage of fused purified pore proteins. Multiple aperture chips can be easily assembled in the 3d-printed holder to conduct parallel membrane transport investigations. Moreover, well defined microfabricated apertures are achievable with very high reproducibility. The presented microsystem allows the investigation of fast gating events (down to 1 ms), pore blocking by an antibiotic, and gating events of small pores (amplitude of approx. 3 pA).

## 1. Introduction

Pores located in the cell membrane of biological species play an important role in controlling cellular activities. The Gram-negative bacterium *Escherichia coli,* for example, uses various porins (channels) for selective nutrients uptake while rejecting toxic substances into the cell [1,2]. OmpF and OmpC (outer membrane proteins) allow such fluxes by passive diffusion of hydrophilic molecules [3,4,5,6,7]. Gram-negative bacteria contain lipopolysaccharides (LPS) on their outer membrane. These chains are responsible for the structural integrity of the bacteria [8,9,10,11]. Ion transport across the membrane of mammalian cells are often investigated by patch clamping [12,13,14,15]. This electrophysiological method cannot be applied on bacteria because these species are much too small to allow patch-clamp recording. Additionally, the LPS layer prevents a proper pipette sealing on the bacterial cell membrane [9,10].

Outer membrane vesicles (OMVs), small particles of approximately 20 nm to 250 nm in diameter, released from the outer cellular wall of Gram-negative bacteria i.e., *Escherichia coli*, *Pseudomonas aeruginosa*, *Chlamydia trachomatis*, *Yersinia pestis*, act as a carrier to transport virulence factors, bacterial proteins, and toxins between bacteria and other biological species. Bacterial survival largely depends on the cell-to-cell communication through OMVs [16,17,18,19,20]. Unlike patch clamping, lipid bilayer membranes (BLM) free standing in a hydrophobic aperture are very suitable to electrically investigate OMV-BLM interactions [21]. A main advantage is that both sides of the spanned lipid bilayer are accessible [22]. This allows titration or exchange of liquid components (such as the ion composition, pH, or antibiotic candidate concentration) on both sides of the membrane. Porins present in OMVs, fused into a BLM, remain in their native lipid and LPS environment. This is a more in vivo-like model compared to purified porins fused into a BLM made from a synthetic mixture of lipids [8]. A native lipid environment is important for maintaining the protein’s structure and its function. Besides applying OMVs, it is also possible to reconstruct membrane proteins in synthetic polymer discs with native lipids [23]. Other potential methods to fuse proteins and membranes are described in [24,25]. Upon successful membrane formation, pore forming porins (e.g., OmpF, OmpC, Gramicidin and alpha-hemolysin) introduced in the liquid reservoir at one of the two membrane sides, can be fused into the BLM. When a DC voltage is set between the two fluid reservoirs, current steps indicate the opening and closing events of the ion channels.

The quality (mechanical stability, resistance and capacitance values [26]), functionality, reproducibility, and preparation time of a BLM are important parameters for ion channel translocation investigations. By painting lipid solution (lipids dissolved in an organic non-polar solvent, e.g., n-decane) on the micro-aperture suspended in an electrolyte solution, the phospholipid molecules naturally arrange with its hydrophilic head group positioned towards the liquid phase and the hydrophobic chain oriented towards the lipophilic aperture. This results in a lipid bilayer membrane containing an excess in solvent that assures a mechanically stable connection with the aperture surface (solvent annulus: Plateau-Gibbs border) [27,28,29]. Unfortunately, inhomogeneous distribution of excess solvent causes solvent-lipid clogs, preventing the required thinning of the membrane (more than two single lipid monolayers), which inhibit the reconstitution of channel-forming membrane proteins. How to construct a high quality and thin BLM that does not contain excess solvent was first introduced in [30]. In this method, lipid-containing solvent is dispensed in chambers filled with electrolyte. Here, the liquid levels are set below the vertically positioned aperture. On top of the liquid, a monolayer of lipids is formed, where the head groups are attracted to the fluid phase and the hydrophobic tail groups are faced outwards (air). After the solvent has evaporated, the lipid monolayers are folded to the aperture by increasing the buffer volume of both reservoirs. Drawbacks of this method are that it requires large amounts of lipid, and that it contains time-consuming solvent evaporation steps [31]. As a promising alternative, stroking an air bubble covered with lipid in the liquid reservoir near the aperture, a BLM can be formed. This method requires the tip of a pipette precoated with lipid solution (lipids dissolved in n-octane). Due to capillary forces, the lipids attach (the hydrophobic tails) to the inner wall and outer wall of the pipette tip. By introducing this air-filled lipid precoated tip in the aqueous solution close to the aperture and stroking the pipette, a lipid-covered air bubble is released near the aperture. This bubble eventually bursts on the aperture wall, creating a thin free-standing BLM [32,33].

A method to realize apertures for BLM construction is by punching holes in Teflon foil [31]. However, mechanical punching yields apertures that are non-planar, thus effecting BLM formation. A more suited method to yield smooth apertures is to apply sparks to melt holes in the Teflon foil [34]. As Teflon is lipophobic, a pretreatment step with non-volatile organic solvent (such as n-hexadecane 1% *v*/*v* in hexane) and a drying step of typically 15 to 20 min is recommended [35]. Furthermore, the integration of Teflon sheets, including small micro-apertures (<100 µm in diameter) in a suitable measuring platform, is often prone to misalignment issues. Instead, silicon-based microfabrication techniques could be well utilized to achieve highly reproducible, small, and well-defined mechanically rigid apertures [36,37]. A material commonly used for the realization of thin membranes in bioelectronics is silicon nitride. Silanization of silicon nitrate structure can be applied to optimize the interaction with biological particles [38]. Treatment with APTES (aminopropyl-triethoxysilane) or PTES (propyl-triethoxysilane), for example, will respectively result in a surface that is hydrophilic or hydrophobic [39,40].

Micro-apertures fabricated in a silicon substrate enhances electrical noise due to the high dielectric loss. This significantly affects signal resolution when conducting BLM translocation experiments. To reduce this noise, silicon substrates are usually coated with dielectric materials such as silicon dioxide, CYTOP or Teflon. Oshima et al., reported on a Teflon-coated aperture to realize a synthetic BLM [41]. Despite the low noise characteristics of the membrane current, the aperture required time-consuming pretreatment steps to make the surface lipophilic. Another polymer, CYTOP, was also tested on silicon nitride membranes [36]. However, CYTOP is not sufficiently lipophilic (the contact angle of a dispensed lipid droplet on a CYTOP-coated surface measures 30°) [42]. Since CYTOP is an amorphous fluoropolymer like Teflon [43], it can be assumed that pre-treatment steps with organic solvent may improve its lipophilic property. Because of the tapered aperture layout obtained by isotropic etching of the silicon nitride membrane, the formation of cracks was observed [36]. Other groups came up with the idea of excluding the main substrate materials (such as glass or silicon). Instead, they relied on bare photoresist or polymer sheets. For example, Baker et al. reported on a tapered SU-8-based micro-aperture realized through complex fabrication steps where rotational UV exposure was employed to improve the mechanical stability of the aperture [44]. Suzuki et al. introduced a thin Parylene sheet consisting of micro-aperture arrays placed in a 3d-printed housing [45]. One of the fabrication steps includes the removal of a fragile thin Parylene sheet from a base substrate and precise alignment with the housing. Due to the horizontal placement of the platform, air pockets were easily formed in the enclosed fluid chamber below the aperture, making BLM measurements very challenging. Teflon is a suitable substrate for forming BLMs to conduct ion-channel measurements. However, Teflon also has certain limitations like aperture miniaturization challenges and difficulties to incorporate the thin and flexible Teflon sheet in an array-based measurement platform. To resolve the above-mentioned issues, we have presented a Parylene-coated silicon/silicon nitride chip where a thin BLM can be quickly formed without any required time-consuming pre-treatment steps [22,33,46]. Two types of Parylene (Parylene-C and Parylene-AF4; a comparison between the two materials is given in the Supplementary Materials) were utilized. Unlike CYTOP or Teflon, conformal CVD deposition of Parylene is possible to ensure a hydrophobic and lipophilic encapsulation around the aperture. By employing the pseudo-painted air-bubble technique, a thin membrane can be formed within a few seconds. Initial measurements showed that a membrane can be constructed within a few seconds, and that OmpF fusion was successful.

In this work, the capability of the Parylene-coated microapertures is presented by performing sensitive membrane translocation. Besides BLM construction by repeated membrane formation-and-breaking experiments, cleaning procedures, the ease of chip assembly, the time to form a thin BLM, and the fusion of pore proteins and outer membrane vesicles into the BLM, have been investigated. The performance of the platform was investigated by conducting biological reference measurements with fast gating OmpF (events faster than 1 ms), small amplitude gating Gramicidin (current steps of less than 4 pA), and the fusion of OMVs released from *E. coli*. The lipid bilayer membrane with fused pore proteins realized in a Parylene-coated aperture functions as a biological sensing unit. Such systems are very suitable for membrane translocation measurements such as antibiotic candidate screening and DNA sequence analysis [47,48].

## 2. Materials and Methods

### 2.1. Sample Preparation

Outer membrane vesicles (OMVs) were prepared from *E. coli* bacteria BL21(DE3)omp8 [49]. This strain lacks the four major outer membrane proteins OmpA, OmpF, OmpC, and LamB. However, from plasmid pGOmpF, OmpF is overexpressed in the *E. coli* bacteria [50]. OMVs were purified by employing differential centrifugation. Based on densitometry measurements on a Coomassie-stained SDS-PAGE gel, the measured final OMV concentration was 10 µM. The average vesicle diameter of 95 nm was determined by dynamic light scattering. A detailed purification protocol is given in [8]. A 1 mg/mL OmpF stock solution was prepared from the OmpF overexpressing *E. coli* stain and diluted 10-fold in Genapol X-080 (1% *v*/*v*) [22] (the influence of the detergent on the BLM is shown in the Supplementary Materials, Figure S5). Gramicidin D (Sigma-Aldrich, Taufkirchen, Germany), an antibiotic that can form a small ion channel when two monomers are aligned at both sides of the bilayer, was diluted in ethanol at a final concentration of 1 mM.

### 2.2. Micro-Aperture Fabrication and 3d-Printed Platform

**Figure 1:**
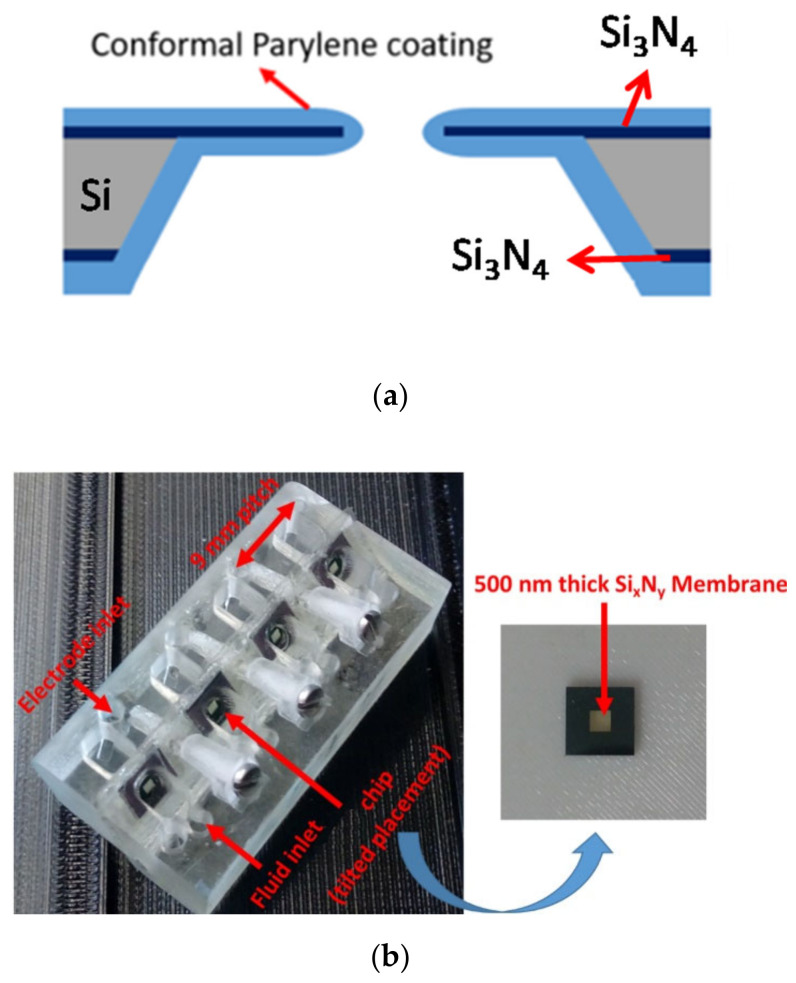

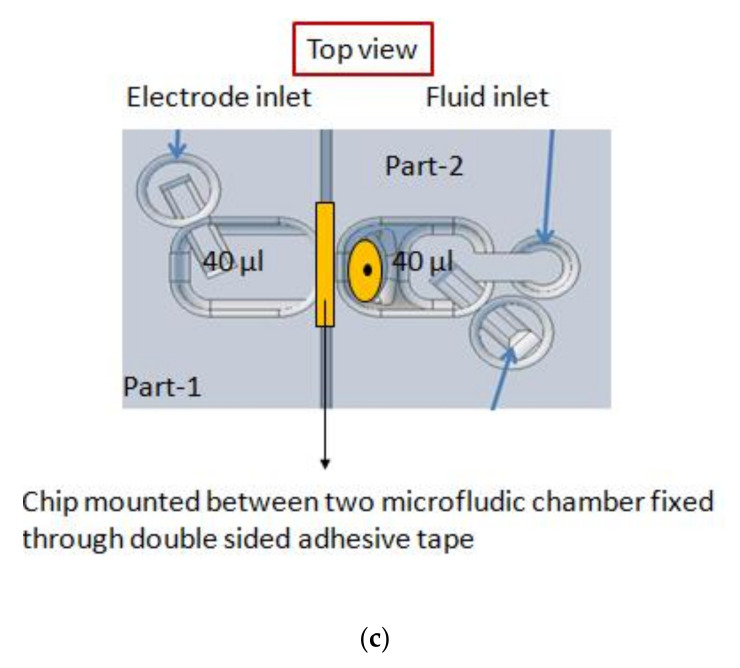
Parylene-coated apertures. (**a**) Parylene-coated silicon nitride aperture (**b**) A photo of an aperture platform with four aperture chip positions. Each chip has its own integrated fluid reservoirs and electrode positions. The distance between the reservoirs is 9 mm, which is compatible with a multichannel pipetting robot. (**c**) A schematic with a chip (yellow) mounted between the 3d-printed holder design.

The fabrication steps of the BLM aperture were already reported in our previous work [22,45,51]. In short, a 380-µm-thick silicon double-polished wafer was introduced in a low-pressure chemical vapor deposition (LPCVD) system to obtain a 500-nm-thick silicon nitride layer. Afterwards, photolithography and reactive ion etching (RIE) was carried out to transfer the aperture and etch profile at the front and backside of the wafer. The aperture (30 µm to 100 µm) was opened by conducting KOH (30%) anisotropic wet chemical etching, where silicon was etched though. Finally, conformal Parylene coating (9.2 µm Parylene-C or 3.5 µm Parylene-AF4) was performed in a Labcoater series 300 (Plasma Parylene Systems GmbH, Rosenheim, Germany). In Figure 1a, a schematic of the processed chip is shown.

**Figure 2:**
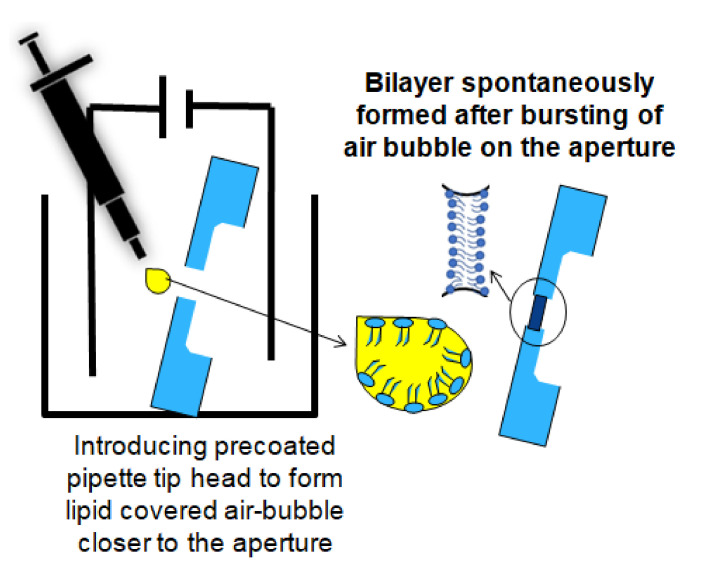
A cartoon of the pseudo-painted air bubble method for BLM formation.

The diced aperture chips were placed in a 3d-printed platform with fluid reservoirs at both sides of the chip. This allows easy access to both the cis and trans side of the BLM, which is required for BLM formation and OMV or pore protein fusion. A render of the sample assembly is depicted in Figure 1c. Autodesk Inventor was used to design the holder. By placing the aperture chips in a tilted position (approx. 45°), it is easy to obtain pore protein and vesicle fusion in a constructed synthetic BLM (pseudo-painted membrane formation is shown in Figure 2; in Supplementary Materials Figure S1, a BLM constructed by conventional painting and pseudo painting is depicted). It also prevents the formation of air pockets around the BLM, which often happens when conducting experiments with horizontal BLM configurations. Each holder accommodates up to four chips and eight fluid chambers with a volume of 40 µL each. The chips are fixed between these 3d-printed holder blocks by applying double-sided medical grade adhesive tape (Figure 1b). The fluid chambers have individual inlets for fluid and electrodes, required for conducting separate electrophysiological experiments. The holder was printed using an Asiga MAX X27 3d-printer with a 385 nm LED light source. A semitransparent photocurable resin (PlastCLEAR) was used to print the holder pieces. After exporting the Inventor model, the design was sliced into individual layers that are projected on the printer platform by UV exposure. After printing, the realized structure was rinsed with isopropanol to remove unexposed resin within the structure, dried with nitrogen gas, and exposed to UV light for five minutes to cross-link the remaining uncured resin at the outside of the print [52].

The chips can easily be reused by cleaning the aperture surroundings and the two reservoirs with ethanol and deionized water.

### 2.3. Formation of Free-Standing Bilayers

Bilayer lipid membranes of DPhPC (1,2-diphytanoyl-sn-glycerol-3-phosphocholine, (Avanti Polar Lipids) dissolved in n-octane at a concentration of 5 mg/mL, were constructed on the Parylene-coated silicon nitride apertures by applying the lipid-coated air bubble bursting technique [32]. The most stable membranes are usually obtained with lipid mixtures (for example, azolectin, egg lecithin). Those lipid mixtures do not have a phase transition in the experimental range. As the composition varies from batch and source, it is advisable to work with synthetic pure lipid: DPhPC. This is fully saturated, and due to four methyl sidechains, always in the fluid phase [53]. Also, there was no report of different results caused by the use of this lipid with respect to more biologically relevant lipids. A free-standing bilayer membrane might restrict fast and reproducible pore protein fusion due to the presence of solvent clogs or the accumulation of multiple lipid layers [32]. Therefore, a procedure is required to verify the quality of the membrane and quickly reform the BLM when necessary. A method to quickly destroy and reform a BLM is by applying a short DC pulse of 1 V (1 s) over the membrane when the BLM has not reached its optimal capacitance range (e.g., for a 90 µm diameter membrane, the range is 36 pF–47 pF) [30,31,54]. Reforming of the membranes can easily be realized by repeating the procedure. (In the Supplementary Materials, Figure S3, repeated construction and destruction of a BLM are depicted to show the quality of the membrane).

### 2.4. Electrophysiological Measurement Method

Electrophysiological measurements were conducted by applying the aperture platform and a current amplifier (eONE, Elements s.r.l., Cesena, Italy) connected to Ag/AgCl electrodes. When a BLM is formed, the current state between the two fluid chambers changes from short-circuited to insulated. The whole system was placed in a metal housing to shield the system for outer electronic disturbances. The membrane capacitance was measured by applying a 200 mV_peak-peak_ triangular wave. The background current noise of a Parylene-C-coated aperture was measured at a sampling-frequency of 10 kHz and a potential of 0 mV, followed by a low-pass filter set at 1 kHz. The same sampling frequency was used during the vesicle (OMVs) and ion channel (OmpF, Gramicidin D) incorporation experiments. OmpF and Gramicidin D gating events were conducted at a DC potential of ±100 mV to ±200 mV, and a sampling-frequency of 10 kHz. OMV fusion was obtained at ±150 mV.

## 3. Results and Discussion

One major advantage of the presented platform is that the rigid predefined microfabricated apertures can easily be positioned in a 3d-printed multichip holder with small (typically 40 µL) volume fluidic reservoirs. The time to assemble a system with four chip apertures takes approximately five minutes. The ease of rapidly forming lipid bilayers is shown where membrane can be stably formed within a few seconds. Moreover, the conformal lipophilic Parylene coating allows membrane formation exceeding the seal resistance greater than 1 GOhm without precoating the aperture (which is often recommended for Teflon-based apertures).

### 3.1. Parylene versus Teflon

The background current noise of the presented BLM platform was compared with a conventional Teflon aperture (approx. 100 µm in diameter). The aperture, punched with a heated wire, was glued between 3d-printed holder pieces (Figure 1b). Experiments with this aperture were carried out by placing the system in a metal housing to reduce external, i.e., electrical and environmental noises.

**Figure 3:**
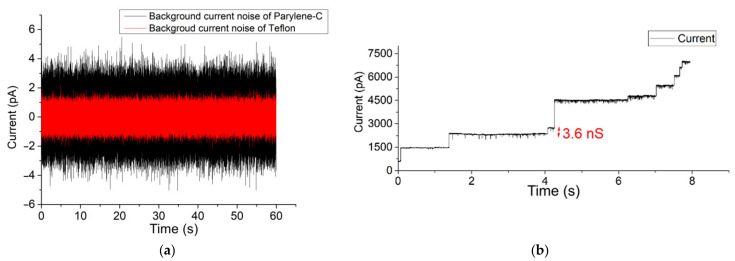

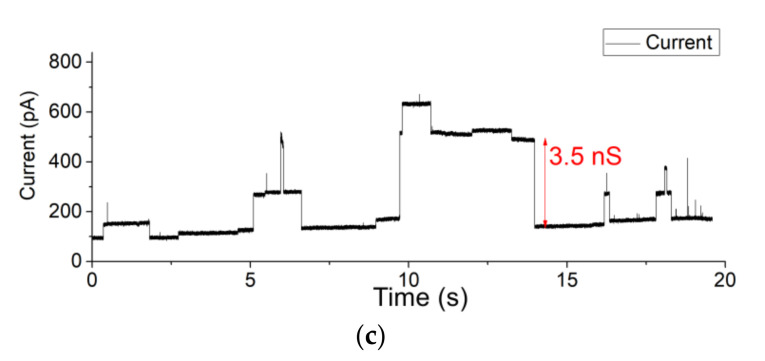
(**a**) Background current noise of Teflon and Parylene-C-coated apertures (**b**) Successful OmpF incorporation in a 100 µm Teflon aperture fixed between reservoirs (**c**) 90 µm Parylene-C-coated aperture system. The applied potential was set to 100 mV. The measurement of BLM incorporated with OmpF was stable for more than 10 min (a larger time span of Figure 3c is given in the Supplementary Materials Figure S8a,b).

After pre-treating the Teflon aperture with n-decane and drying with nitrogen gas, a BLM was created using the pseudo-painting method (Figure 2). In Figure 3a, the maximum peak-to-peak current noise is approximately 3 pA (8-pole Bessel low-pass filtered at 1 kHz; 3-sigma accuracy [55]). Usually, a BLM is stable for more than 25 min. OmpF was introduced in the chamber located at the cis side of the BLM. Multiple gating conductance steps were observed when a 100 mV potential was applied across the membrane. In Figure 3b, the conductance step of the highlighted current step is 3.6 nS. After fusion of OmpF into the BLM (OmpF dissolved in detergent to create fusible micelles), the BLM was stable long enough to conduct gating activity recordings. The BLM used to record the OmpF gating events in Figure 3c was stable for 10 min.

The same potential and filter settings applied over a constructed BLM in a 90 µm diameter Parylene-C-coated silicon nitride aperture resulted in a similar gating conductance value (approx. 3.3 nS) [51]. BLMs constructed in a free-standing Parylene-C-coated aperture were also stable for at least 25 min. Within the highlighted trace, the background current noise has an approximate peak-to-peak current of 7 pA (Figure 3a). This background current noise is due to the silicon substrate, which causes excess noise due to parasitic capacitances. However, this noise level is too high for typical single-channel analysis.

The current-voltage response measurement after OmpF incorporation into a 100 µm diameter Parylene-AF4 chip and 100 µm diameter Teflon aperture is provided in the Supplementary Materials (Figure S7). Here, almost zero leakage current was observed when applying 0 mV over the membrane. The functionality of two Parylene types have been investigated. They are Parylene-C, the most commonly used version, and Parylene-AF4, a fluorinated form with much higher temperature resistance and higher water impermeability properties [56,57]. It is evident that a low background current noise on the membrane is particularly helpful when screening for single-channel activities of small pore-forming ion channels. In Supplementary Materials (Figures S3 and S4), the formation reproducibility of a BLM constructed in a Parylene-coated aperture is depicted.

### 3.2. Fast Gating Measurements of OmpF

**Figure 4:**
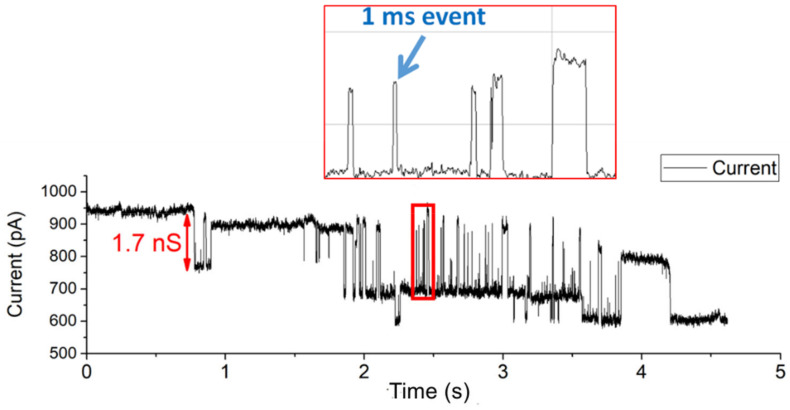
Detection of rapid 1 ms to 3 ms OmpF gating events.

In Figure 3, the depicted voltage-induced gating events of OmpF were all longer than 100 ms. OmpF is known to also gate much faster [58]. The high SNR and ability to show short subconductance events make the conformal Parylene-C-coated apertures very suitable to investigate fast OmpF gating events (ca. 1 ms). An amount of 1 µL of a 1 mg/mL OmpF stock solution was introduced at the cis side of the BLM chamber. Within a few minutes, fast gating events of approximately 1 ms to 3 ms were clearly observed (Figure 4). The signal was recorded while setting the sampling frequency at 10 kHz and applying a 1 kHz low-pass 8-pole Bessel filter. The highlighted 1 ms event has a conductance level of 1.7 nS at 150 mV DC voltage.

**Figure 5:**
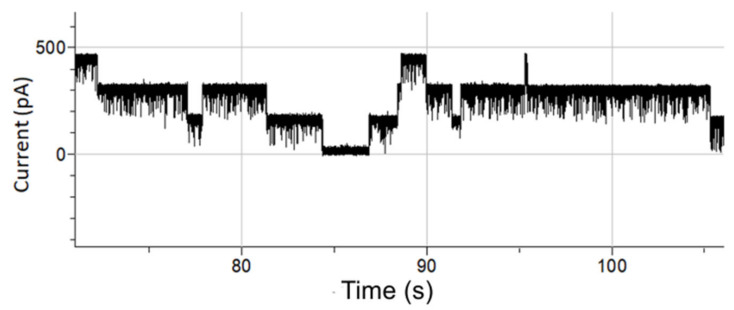
Antibiotic translocation (Kanamycin) through OmpF pores fused in a lipid bilayer. The applied voltage was set to 100 mV DC.

In Figure 5, an OmpF-blocking experiment is shown where 0.5 µL of 10 mM, the antibiotic kanamycin sulphate, was added at the cis side of a lipid bilayer with incorporated OmpF. After applying a 100-mV voltage, sharp spikes resembling the blocking of OmpF pores due to monomeric tunneling of kanamycin, occur.

### 3.3. OMV Fusion in Free-Standing Lipid Bilayer Membranes

**Figure 6:**
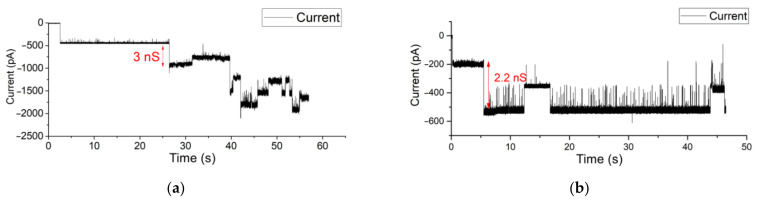
(**a**,**b**). Gating events in a Parylene-C-coated aperture of OmpF incorporated in a BLM by fusion of OMVs. The applied voltage was set to −150 mV (Additional measurements in different chambers are shown in the Supplementary Materials Figure S6).

Instead of fusing purified proteins into a BLM, a more in vivo-like method is to use vesicles released from biological cells containing the protein(s) of interest, surrounded in their native lipid environment [8,59,60]. In Figure 6, measurement results are depicted of a recording where OMVs with OmpF proteins were fused in a free-standing BLM in a Parylene-C-coated aperture chip. The conductance level of such vesicle promoted membrane-inserted OmpF shows conductance levels (2.6 to 3.6 nS) at −150 mV holding potential.

**Figure 7:**
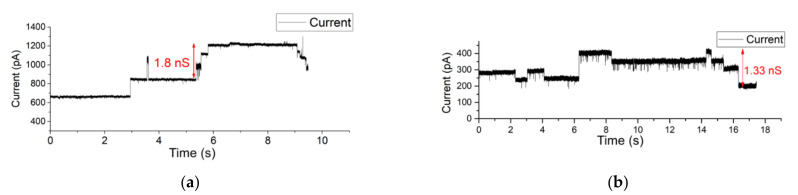
(**a**,**b**) Electrophysiological measurement of OMV incorporation in an 80 µm Parylene-AF4-coated aperture using (**a**) a symmetric 1 M KCl buffer solution (**b**) asymmetric buffer solution (250 mM NaCl at the cis side and 25 mM NaCl at the trans side of the BLM).

Similar measurements were conducted on a Parylene-AF4-coated system with an aperture diameter of 80 µm. Here, OMVs were introduced at the cis side of the BLM. The osmotic fusion of OMVs was stimulated by applying a symmetric buffer solution of 1 M KCl buffer. KCl was used because the electrophoretic mobility of the cation and anion are similar, so that there is no polarization. As a result, upon application of a membrane, potential multiple closing gating events of OmpF pores were observed directly after OMV–BLM fusion occurred within a few minutes. After the fusion of OMVs, the current steps (opening and closing events) of OmpF monomers were observed confirming the successful vesicle and protein incorporation in the BLM (see Figure 7a). The applied holding potential was kept at 200 mV. (In the Supplementary Materials, Figure S8c, a larger timespan of this measurement is depicted).

After the fusion of OMVs, the liquid at both sides of the BLM was exchanged with asymmetric 250 mM NaCl at the cis side and 25 mM NaCl at the trans side of the BLM. As a result, upon application of a membrane, potential multiple closing gating events of OmpF pores were observed directly after OMV–BLM fusion occurred. When applying a holding potential of 150 mV (Figure 7), gating events of OmpF were clearly observed. In Figure 7b, for example, the indicated current step represents a conductance level of 1.33 nS.

**Figure 8:**
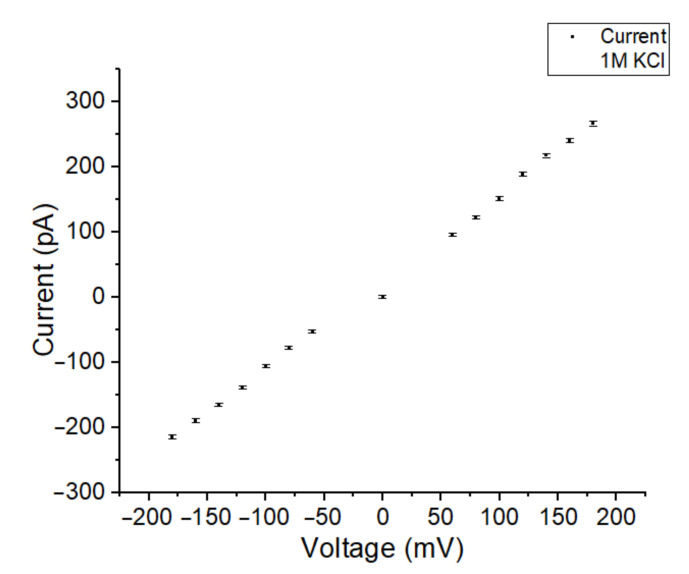
The current-voltage dependency of a BLM after OMV incorporation in symmetric 1 M KCl electrolyte solution. The BLM was spanned in an 80 µm Parylene-AF4-coated aperture.

Upon successful OMV incorporation using a symmetric buffer solution, the formed BLM acts as a conductor that shows a linear relationship with the current at different holding potentials. In Figure 8, the voltage was varied between −200 mV and 200 mV.

### 3.4. Single-Channel Detection of Gramicidin-D Preparation

**Figure 9:**
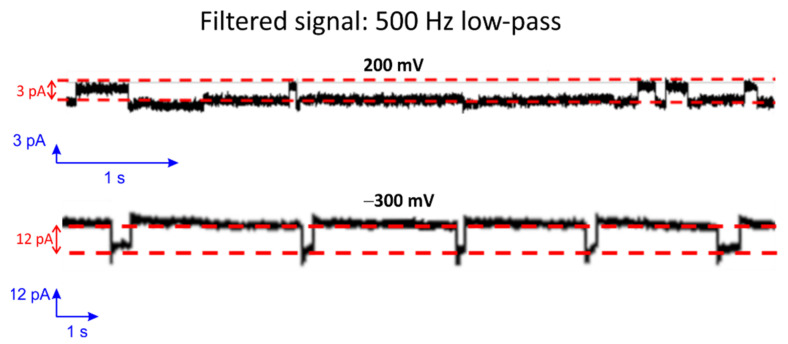
Single-channel detection of Gramicidin D fused in a BLM at a holding potential of 200 mV (**top**) and −300 mV (**bottom**). The unbuffered solution contained 2 M KCl at room temperature.

Gramicidin D (a mixture of A, B and C) is a naturally occurring peptide with antibiotic activity and is commonly used for treating various bacterial infections [61]. When two Gramicidin molecules are aligned from both sides of the bilayer, it occasionally leads to pore formation [62,63,64,65,66,67]. The low single-channel conductance levels resulting through such small pores in Gramicidin are challenging to characterize. In particular, this is because the channel-forming ability of Gramicidin depends strongly on the thickness of the lipid membrane as well as the fluidity and, thus, on the temperature. Therefore, it is often used as a standard for good bilayer formation investigations. To demonstrate the capability of the presented Parylene-coated silicon nitride aperture for small single-channel translocation investigations, Gramicidin D was added at both sides of the BLM in 2 M KCL solution. Figure 9 shows current step events with 0.5 kHz filtering of the signal. The conductance level of such pores amounts to approximately 15 pS at 200 mV and 40 pS at −300 mV. Moreover, the conductance value of the small pore is similar to the reported value found in the literature [41,68].

Although the above-presented Gramicidin D experiments were conducted at room temperature, it is important to know that temperature has a significant effect on the activity and lifetime of Gramicidin. The activity strongly depends on the alignment of two monomers at each side of the bilayer [69,70]. Increasing the temperature enhances the mobility of the monomers [41], but reduces the lifetime of a Gramicidin pore (from 15 s at 10 °C down to 0.01 s at 45 °C [71]). However, the integration of a temperature sensing and heating element can be easily adapted on the chip. This could be a promising future prospect to investigate the effect of temperature variation for Gramicidin experiments in Parylene-coated aperture chips.

## 4. Conclusions

Microfabricated silicon nitride apertures on a silicon substrate were conformal coated with Parylene (types -C and -AF4). These aperture chips were successfully used to investigate translocation of ions through OmpF and Gramicidin pore proteins fused in free-standing lipid bilayers. The hydrophobic and lipophilic properties of Parylene strongly simplifies the handling steps to realize small (<100 µm diameter) free-standing BLMs. Moreover, the exact positioning of these small microfabricated apertures makes it suitable for automated systems. The signal to noise level of the aperture system for both Parylene types are more than sufficient to investigate the fast gating events of OmpF (of approximately 1 ms) and the small amplitude gating of Gramicidin (current steps approximately 3 pA). The Parylene-coated apertures were also successfully used to fuse OMVs, released from *E. coli* overexpressing OmpF. In a following step, the integration of electrodes into the microfabricated aperture chip will be investigated. In addition, a heating element and temperature sensor will be incorporated into the system, allowing the investigation of temperature-sensitive lipid compositions and pore proteins.

## Data Availability

Data sharing is not applicable to this article.

## Supplementary Materials

The following supporting information can be downloaded at: https://www.mdpi.com/article/10.3390/membranes12030309/s1, Figure S1: A bilayer lipid membrane (BLM) realized by conventional painting (left) and by bubble stroking (in a 100 µm Parylene-C coated aperture); Figure S2: (a) Side view and top view of a 100 µm diameter microfabricated Parylene coated aperture. (b) A render of a holder for four aperture chips; Figure S3: (a–c). Zapping of air bubble stroked BLMs, constructed in Parylene-C coated silicon-nitride aperture chips; Figure S4: Repetitive formation of an BLM in an 80 µm Parylene-AF4 coated aperture; Figure S5: Current trace (−100 mV) of a planar bilayer in presence of detergent solution (0.04% GenapolX-080 in 1 M KCl buffer) and the trace was passed through a low-pass filter (1 kHz); Figure S6: (a–c) OMV fusion in multiple chambers (applied voltage: ±150 mV, 10 kHz sampling frequency, no filtering); Figure S7: Current-voltage response of a 100 µm Parylene-AF4 chip (a) and a 100 µm Teflon aperture (b); Figure S8: Extended timeframe of the measurements shown in Figure 3c (a,b) and Figure 7a (c) of the main document. References [30,31,72,73] are cited in Supplementary Materials.
