# Supplementary material for: Silicon Nitride-Based Micro-Apertures Coated with Parylene for the Investigation of Pore Proteins Fused in Free-Standing Lipid Bilayers
Source: Membranes (Basel). 2022 Mar 9;12(3):309. doi: 10.3390/membranes12030309 (PMC8954132; doi:10.3390/membranes12030309)
Supplement: Supplementary file 1 [file membranes-12-00309-s001.zip › membranes-1576984-supplementary.pdf]

membranes

Article

Silicon Nitride-Based Micro-Apertures Coated with Parylene for the Investigation of Pore Proteins Fused in Free-Standing Lipid Bilayers

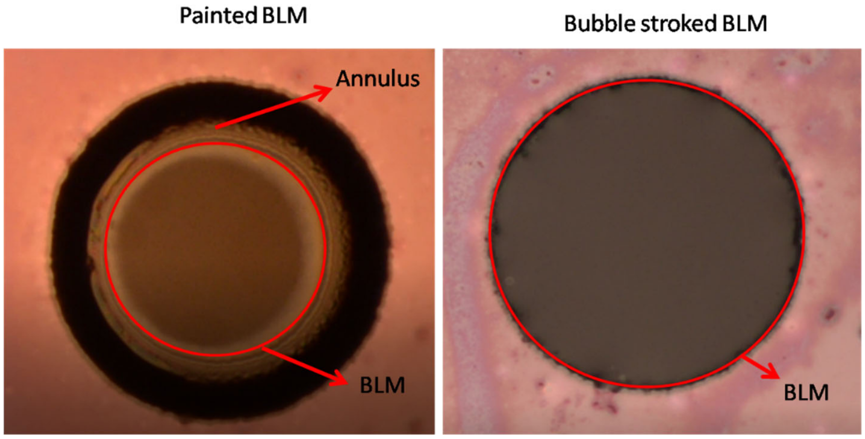

Figure S1. A bilayer lipid membrane (BLM) realized by conventional painting (left) and by bubble stroking (in a 100 μm Parylene-C coated aperture).

Parylene-C vs Parylene AF4

Parylene-AF4 is a fluor based polymer that has in comparison with Parylene-C, a much higher thermal stability (continuous service temperature of up to 350°C, compared to 80°C for Parylene-C), and lower moisture absorption (less than 0.01% after 24 h, compared to less than 0.1% for Parylene-C). Moreover, it has higher penetration ability, making it more suitable to coat the inner structures of small microfluidic structures. This we have shown in a previous work (Akhtar et al., https://doi.org/10.1016/j.snb.2017.08.032), where Parylene coated microfluidic channels were utilizes to store droplets. Both types of Parylene show similar hydrophobic and lipophilic characteristics. Contact angle measurements of dispensed water and lipid droplets on these two coatings are given in our publication (Ahmed et al., https://doi.org/10.1007/s10544-020-0473-y). The contact angle for water and lipid droplets on Parylene-C and AF4 amounts to 92°, 7° and 101°, 3° respectively.

One important advantage of Parylene-AF4 over Parylene-C is that it requires a thinner conformal coating to ensure similar hydrophobic and lipophilic characteristics. The bilayer lifetime (at least 25 min) and stability is the same for both coating types. Since the applied bubble method only introduces a very tiny amount of solvent, without creating a DPhPC lipid and solvent containing ring (the annulus), the BLM area is approximately the size of the aperture. A comparison picture containing a formed BLM by conventional painting and by bubble stroking has been added to the manuscript's Supplementary information (Figure S1). In S2(a,b), a SEM image of Parylene-C coated aperture and a holder design for four aperture chips including liquid reservoirs and electrode inlets is depicted.

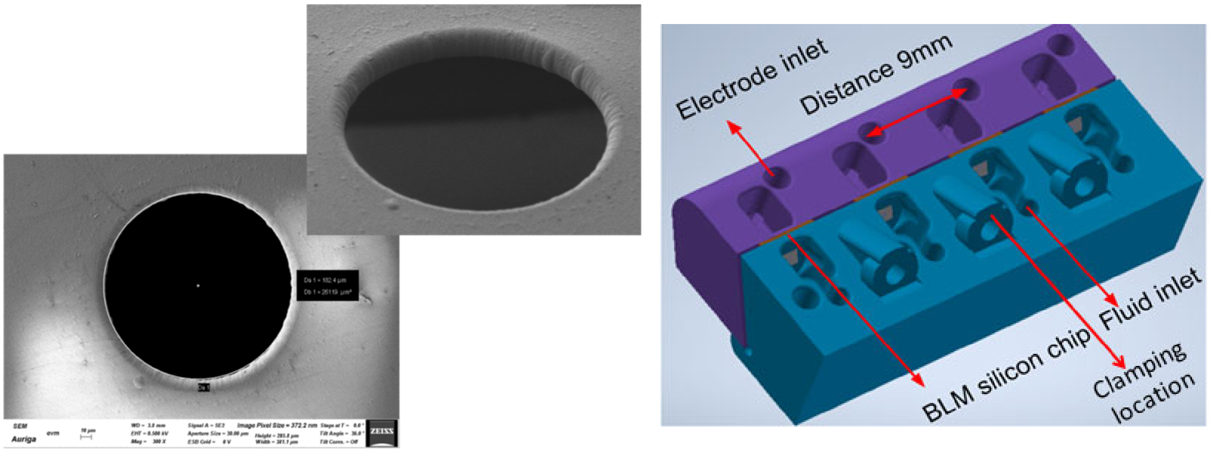

(a) **(b)**

Figure S2. (**a**) Side view and top view of a 100 µm diameter microfabricated Parylene coated aperture. (**b**) A render of a holder for four aperture chips.

Additional measurements

The formation reproducibility of a DPhPC BLM constructed in Parylene coated aperture was investigated by repetitive construction and destruction of the membrane. Destruction of the membrane was achieved by applying 1 Volt DC pulses. It typically takes around thirty seconds to construct a lipid membrane in a Parylene coated aperture. In the following experiment, Parylene-C coated chips with a 90 µm diameter aperture were fitted in the 3d-printed chip holder. The measurement results are shown in FigureS3(a–c).

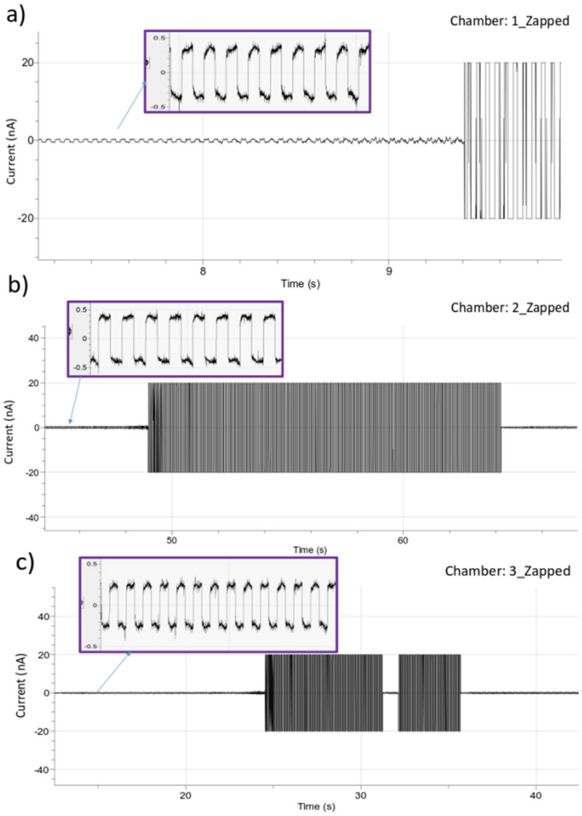

Figure S3. (**a–c**). Zapping of air bubble stroked BLMs, constructed in Parylene-C coated silicon-nitride aperture chips.

The recorded BLM capacitances for these experiments were 36 to 43 pF. From the capacitance value, the hydrophobic core thickness was derived (hydrocarbon tail length of the lipid molecules) of the BLM. The membrane can be considered as a parallel plate capacitor [1]. The measured capacitance value yields a thickness of the phospholipid tails of approximately 2.5 nm. This results in a capacitance per unit area (Cs) between 6.8 fF/µm2 to 9 fF/µm2. Similar experiments were conducted on Parylene-AF4 coated apertures with a diameter of 80 µm (FigureS4).

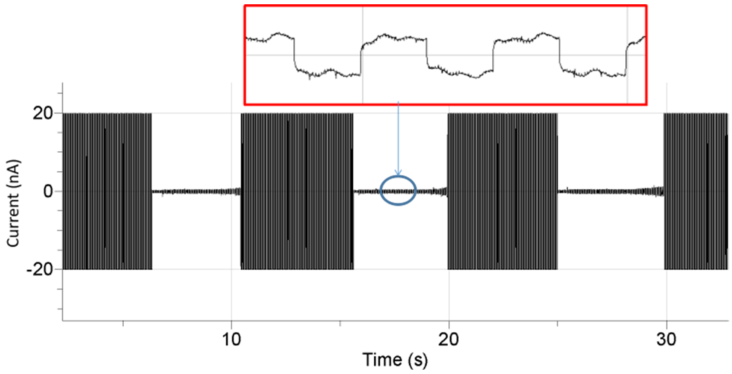

Figure S4. Repetitive formation of an BLM in an 80 µm Parylene-AF4 coated aperture.

The measured BLM capacitance of 48 pF was similar to the measured Parylene-C coated apertures. The resultant capacitance per unit area (Cs) amounts to 9.8 fF/µm2, which is in the same range as found by [2–4]. The repetitive BLM construction and destruction experiments on both Parylene systems confirm the formation of free-standing thin bilayer membranes that are suitable for electrophysiological measurements of incorporated OMVs and ion channel proteins.

Figure S5 depicts a negative control experiment where detergent (Genapol X-080) was added after forming the lipid bilayer membrane in a 90µm Parylene-C coated aperture. −100 mV was applied to record the resultant current (sampling frequency: 50 kHz, bandwidth: 10 kHz, low-pas filter at 1 kHz; the same parameters were applied during the OmpF inhibitor Kanamycin translocation experiment). The small current drift (highlighted in the insert) indicates that the membrane is slightly solubilised due to the added detergent.

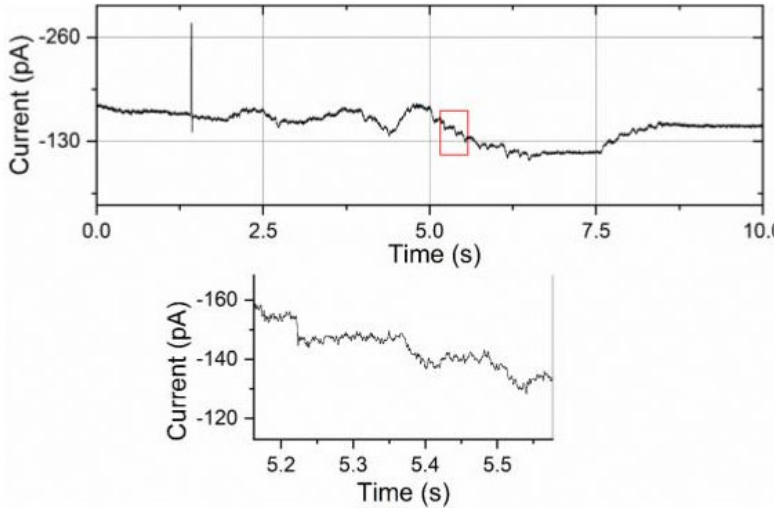

Figure S5. Current trace (−100 mV) of a planar bilayer in presence of detergent solution (0.04% GenapolX-080 in 1 M KCl buffer) and the trace was passed through a low-pass filter (1 kHz).

Multiple OMV fusion experiments were conducted at once. However, only a single amplifier was applied. By repositioning the electrodes in the multi-chip holder during the measurement, multiple bilayers were investigated. In figure S6, the recordings of three chambers are given.

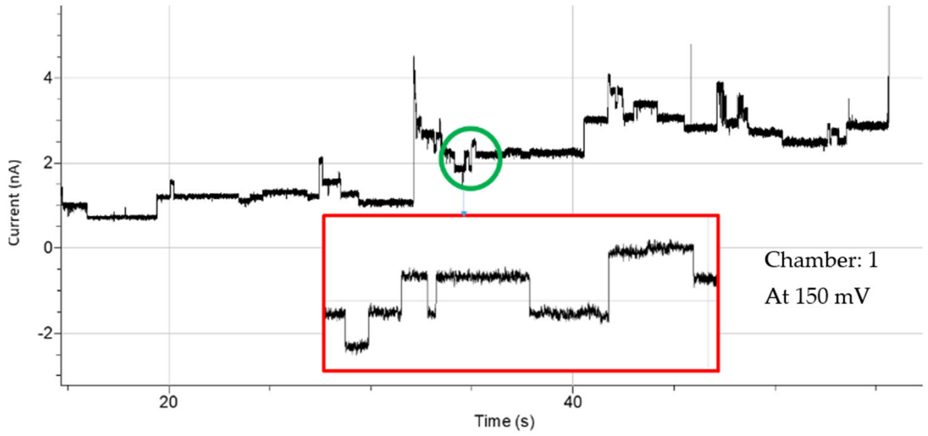

(**a**)

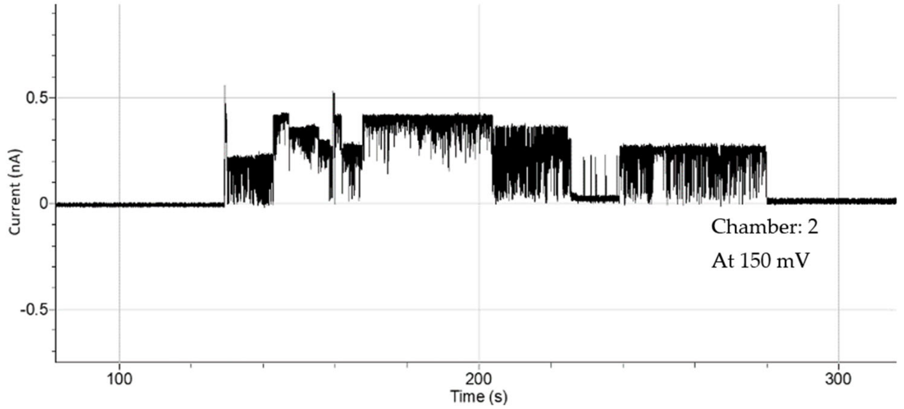

(**b**)

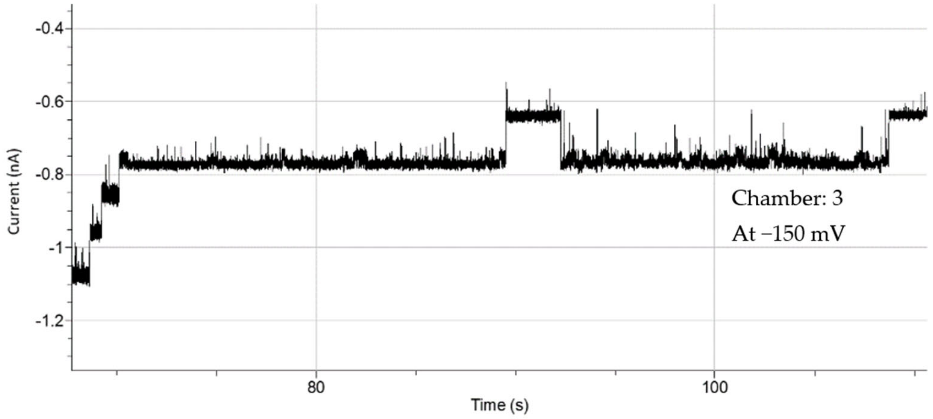

(**c**)

Figure S6. (**a**–**c**) OMV fusion in multiple chambers (applied voltage: ±150 mV, 10 kHz sampling frequency, no filtering).

The current-voltage response of a 100 µm Parylene-AF4 chip (Figure S7a) and a 100µm Teflon aperture (Figure S7b) in a 1 M KCl solution was recorded after successful OmpF incorporation into the BLM (sampling frequency: 50 kHz, bandwidth: 10 kHz).

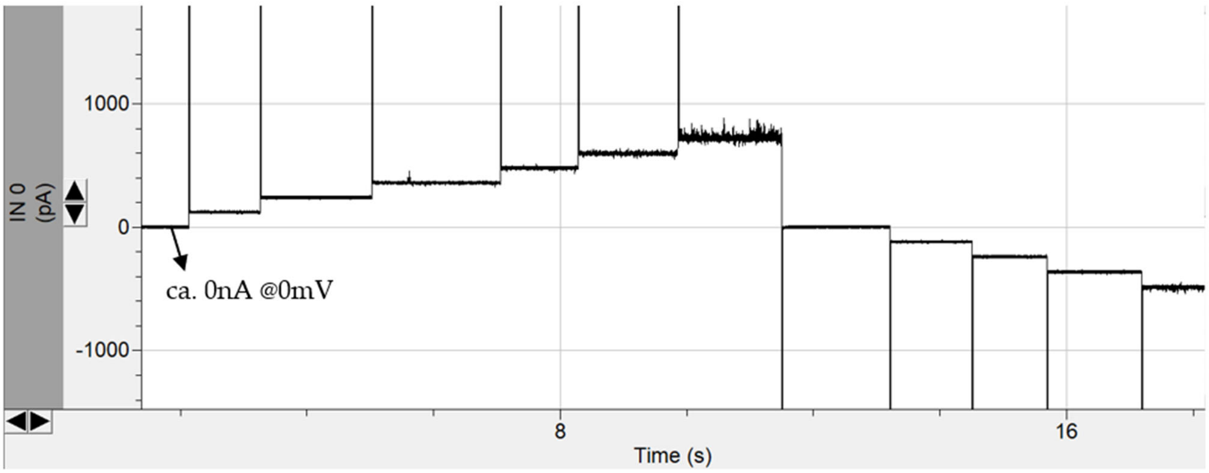

(**a**)

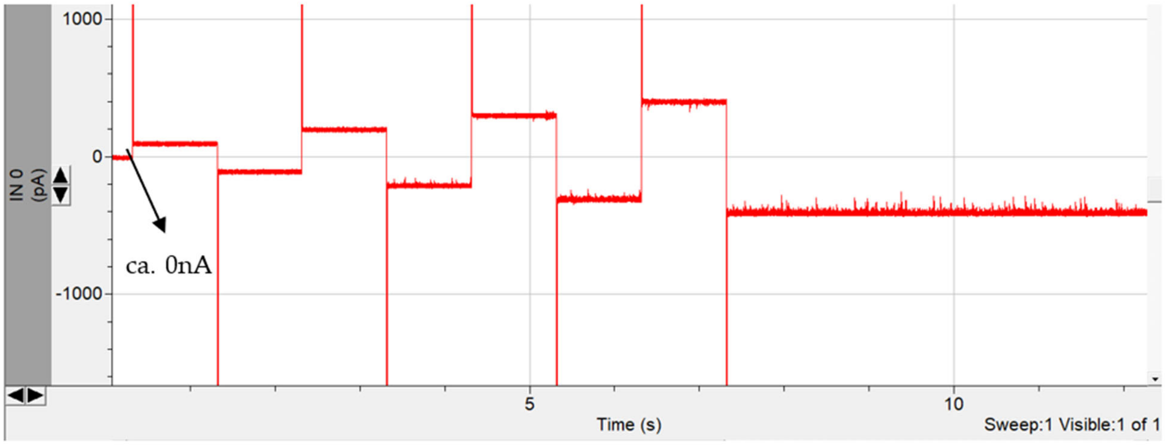

(**b**)

Figure S7. Current-voltage response of a 100µm Parylene-AF4 chip (**a**) and a 100µm Teflon aperture(**b**).

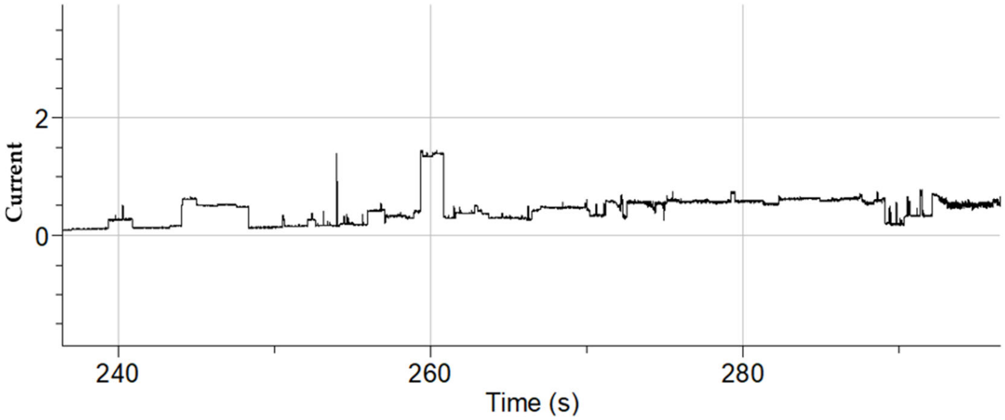

(**a**)

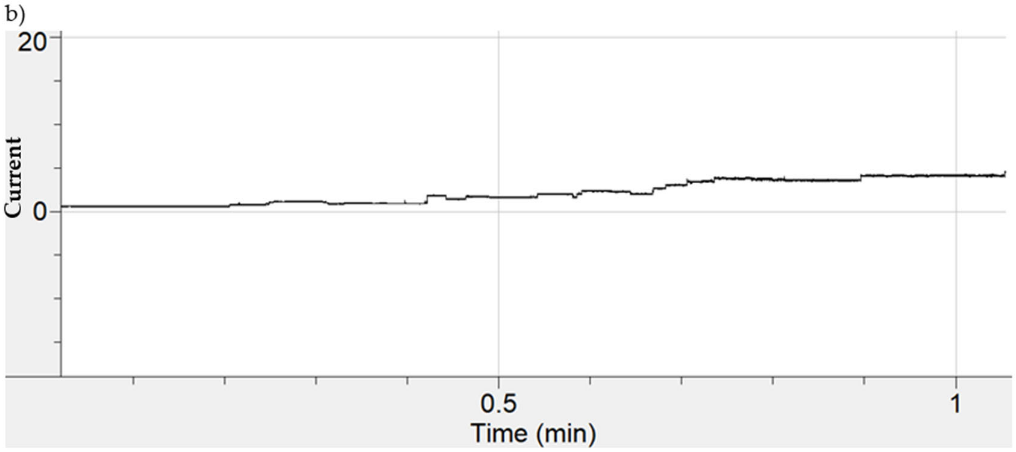

(**b**)

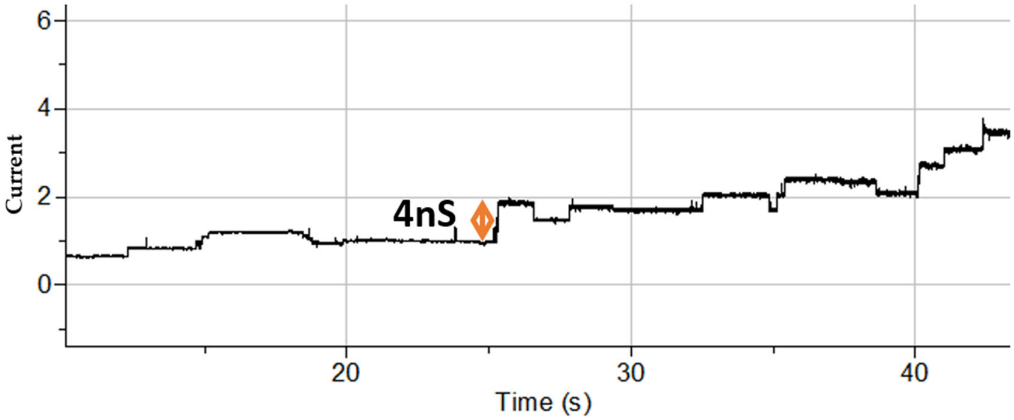

(**c**)

Figure S8. Extended timeframe of the measurements shown in Figure 3c (**a**,**b**) and Figure 7a (**c**) of the main document.

References

1. Hille, B. Ionic channels in excitable membranes. Current problems and biophysical approaches. *Biophys. J.* **1978**, *22*, 283–294, doi:10.1016/S0006-3495(78)85489-7.
2. Montal, M.; Mueller, P. Formation of Bimolecular Membranes from Lipid Monolayers and a Study of Their Electrical Properties. *Proc. Natl. Acad. Sci.* **1972**, *69*, 3561–3566, doi:10.1073/pnas.69.12.3561.
3. Hanke, W.; Schlue, W.-R. *Planar Lipid Bilayers*; Elsevier: Amsterdam, The Netherlands, 1993; ISBN 9780123229953
4. Ridi, A.; Scalas, E.; Robello, M.; Gliozzi, A. Linear response of a fluctuating lipid bilayer. *Thin Solid Films* **1998**, *327–329*, 796–799, doi:10.1016/S0040-6090(98)00789-5.
